# Preoperative mental illness and postoperative atrial fibrillation in cardiac surgery patients: Identifying a vulnerable population

**DOI:** 10.1017/cts.2022.493

**Published:** 2022-11-15

**Authors:** Natalie K. Kolba, Byungho Lee, Henry J. Tannous, Thomas V. Bilfinger, Annie L. Shroyer

**Affiliations:** 1 Department of Surgery, Stony Brook University, Stony Brook, New York, USA; 2 Division of Cardiothoracic Surgery, Stony Brook University, Stony Brook, New York, USA

**Keywords:** Atrial fibrillation, cardiac surgery, mental illness, depression, anxiety, risk factors, comparative effectiveness research

According to the American Psychiatric Association, mental illnesses represent conditions where the emotions, thinking, or behavior of patients are altered. Given the close association between mental illness and stress, an imbalance of neurotransmitters is often a driver for the pathophysiologic changes observed [1]. For example, patients with mental illnesses have been reported to have a higher prevalence of cardiovascular disease, which may be partially due to lifestyle-based risk factors [2, 3]. It has been found that both mental illnesses and their associated risk factors, including delays in care and medications, contribute to other postoperative outcomes such as a higher readmission rate, more non-fatal cardiac events, repeat cardiac procedures, and overall higher mortality rates [4, 5]. When mentally ill patients are considered for surgery, their cardiac status is difficult to optimize because of the additional complications. Additionally, patients with cardiovascular disease often have concomitant conditions such as renal failure and diabetes [6]. Because of these comorbidities, it is difficult to attribute any post-surgical complications purely to a mental illness diagnosis without additional statistical analysis. However, publications reporting preoperative risk factors have passed over the influence of mental illness diagnoses on clinical outcomes and resource utilization.

Given the paucity of articles identified using traditional search techniques, a very broad MEDLINE (PubMed) database search combined with manual screening of all articles was undertaken. For the 81 relevant articles found with multivariable risk models predicting new-onset atrial fibrillation, a wide variety of patient risk factors were reported; however, only 2 even considered the potential influence of mental illness. Katznelson *et al.* [7] prospectively observed 107 coronary artery bypass graft (CABG) patients to identify if preoperative depression was associated with new postoperative arrhythmias. Arrhythmias were assessed based on postoperative Holter monitoring. Comparing depressed versus non-depressed CABG patients, the new postoperative arrhythmias rates were not different (37.9% vs 35.9%: *P* = 0.50). Upon multivariable analysis, older age – but not depression – was identified as the most important risk factor impacting new onset of arrhythmias [7].

In Australia, Tully *et al*. [8] studied 226 CABG patients. By the fifth day post-surgery, 56 (24.8%) of the CABG patients developed new-onset postoperative atrial fibrillation (POAF). Arrhythmias were detected via Holter monitor and daily electrocardiograms. Psychological assessments were based upon the three Depression Anxiety Stress Scales (DASS) where clinically relevant symptoms were identified for depression, anxiety, and stress. Although baseline psychological assessments did not predict POAF, patients’ postoperative DASS-identified anxiety had increased POAF odds (OR 1.09; 95% CI 1.00, 1.18; *P* < 0.05) [8].

To summarize the correlation of preoperative and postoperative mental illness with postoperative atrial fibrillation, odds ratios were identified through a forest plot (Fig. 1). The forest plot supports that patients who developed atrial fibrillation were more likely to have mental illness compared to the non-atrial fibrillation group.

**Fig. 1.**
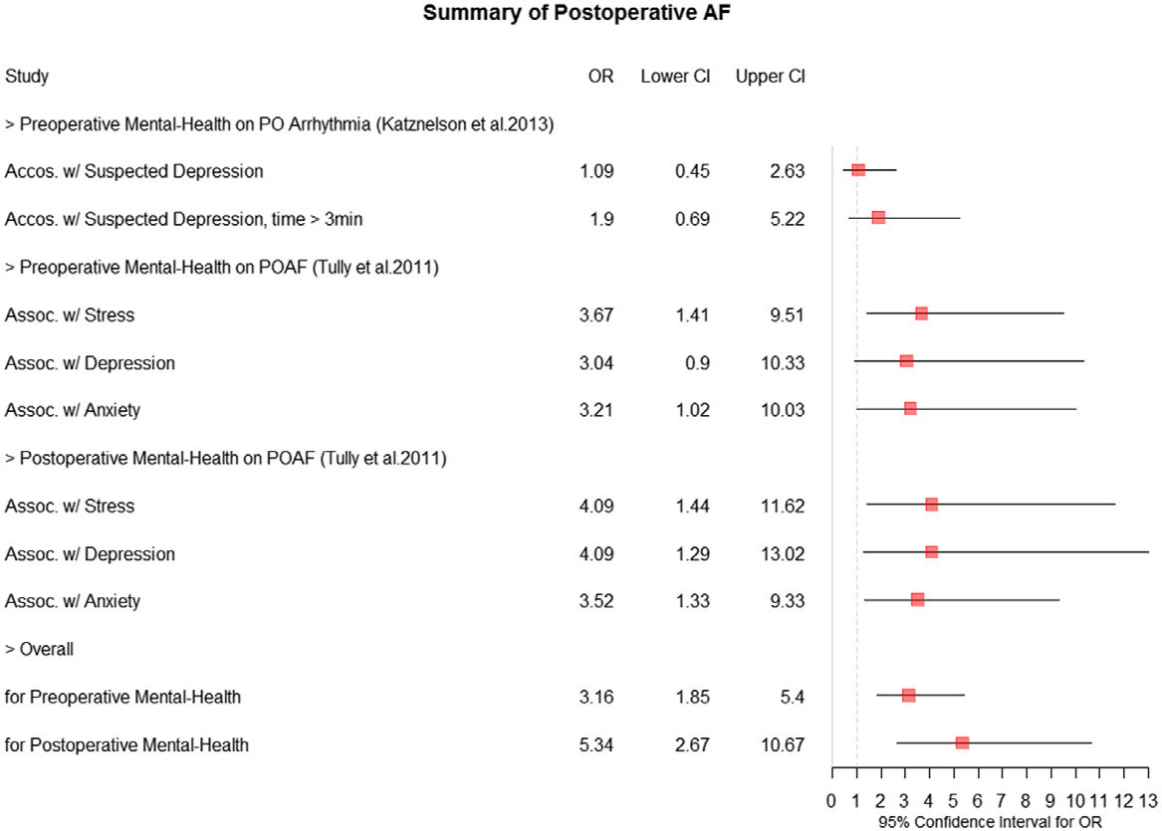
Forest plot summary of postoperative atrial fibrillation (AF). CI = confidence interval, PO = postoperative, POAF = postoperative atrial fibrillation.

In general, mentally ill patients have been under-represented in research but are overall shown to receive less medical care and suffer from more complications [9]. In fact, cardiovascular disease is the leading cause of death in patients with severe mental illnesses. One of the major reasons for this is related to the higher prevalence of smoking, obesity, lack of exercise, and alcohol consumption in these patients [10]. Patients with less medical care are also less likely to undertake testing such as cholesterol screening, which is related to higher rates of undiagnosed cardiovascular disease [9]. Gender and ethnicity may also play a role. Studies showed females are more likely to be diagnosed with a mental illness and cardiac conditions are associated with anxiety and depression among groups such as Caribbean Blacks [11, 12]. Unfortunately, even taking antidepressant and antipsychotic medications can have cardiovascular side effects and symptoms that need to be considered while prescribing [13]. These medications can alter signaling pathways and lead to arrhythmias, especially in patients with other risk factors [14].

Given the potential for a mental illness impact, it is disconcerting that large [e.g., the Society of Thoracic Surgeons (STS)], adult cardiac surgical databases do not gather enough data regarding cardiac surgery patients’ preoperative mental illness. Further, there is little known about how to manage cardiac surgical patients’ psychiatric medications peri- and postoperatively. Of note, medications such as anti-depressants are potent cytochrome inhibitors, risking postoperative drug-drug interactions [15, 16]. Thus, “at risk” mentally ill cardiac surgical patients provide a unique challenge to cardiac treatment selection, perioperative care management, as well as to cardiac research enrollment decisions. Finding innovative, patient-centered approaches to engaging more actively mentally ill patients with concomitant cardiac disease to participate in future research investigations now appears warranted to improve this vulnerable patient population’s overall cardiovascular health and quality of life.

## Data Availability

Not applicable.
